# Validation of a diabetes numeracy test in Arabic

**DOI:** 10.1371/journal.pone.0175442

**Published:** 2017-05-04

**Authors:** Hussah Alghodaier, Hoda Jradi, Najwa Samantha Mohammad, Amen Bawazir

**Affiliations:** 1 King Abdullah International Medical Research Center, King Saud Bin Abdulaziz University for Health Sciences, College of Public Health and Health Informatics, Department of Community and Environmental Health, Riyadh, Saudi Arabia; 2 Al-Faisal University School of Medicine, Riyadh, Saudi Arabia; Weill Cornell Medical College Qatar, QATAR

## Abstract

**Background:**

The prevalence of diabetes Mellitus in Saudi Arabia is 24%, ranking it among the top ten Worldwide. Diabetes education focuses on self-management and relies on numeracy skills. Poor numeracy may go unrecognized and it is important to have an assessment tool in Arabic to measure such a skill in diabetes care.

**Objectives:**

To validate a 15-item Diabetes Numeracy Test (DNT-15) in the Arabic Language as a tool to assess the numeracy skills of patients with diabetes and to test its properties among Saudi patients with diabetes.

**Methods:**

A 15-question Arabic-language test to assess diabetes numeracy among patients with diabetes on the basis of the diabetes numeracy test (DNT-15) was validated among a sample Arabic speaking Saudi patients with diabetes. Data collection included patients’ demographics, long-term glycemic control, diabetes type, duration, co-morbidities, and diabetes related knowledge questions. Internal reliability was assessed using Kuder-Richardson Formula 20 (KR-20).

**Results:**

The average score of Arabic DNT-15 was 53.3% and took an average of 30 minutes to complete. The scores significantly correlated with education, income, HbA1c, and diabetes knowledge (p<0.05). Content Validity Ratio (CVR) of 0.75 and Content Validity Index (CVI) of 0.89 supported good content validity. The Arabic DNT-15 also had good internal reliability (KR20 = 0.90).

**Conclusion:**

Patients with diabetes need numeracy skills to manage their disease. Level of education does not reflect level of numeracy, and low numeracy skills might be unnoticed by health care providers. The Arabic DNT-15 is a valid and reliable scale to identify Arabic speaking patients with difficulties in certain diabetes-related numeracy skills.

## Introduction

Diabetes is one of the most common metabolic disorders in the world. The morbidity and mortality associated with diabetes are substantial, causing a tremendous burden on health and the economy. Saudi Arabia has the seventh highest prevalence of diabetes in the world (24%).^(1)^ Saudi Arabia also has one of the highest incidences of type 1 diabetes (T1D) among children below 14 years of age [1].This serious public health issue should be addressed using all means available.

Patients with diabetes need certain skills to be able to handle their disease effectively and thereby avoid unnecessary complications. In particular, these patients face many mathematical challenges and rely on numeracy skills for the daily self-management of their health, such as to administer insulin, to interpret glucose meter readings [2]. The concept of health numeracy has emerged in the healthcare community [3] as an indicator that would help healthcare providers to communicate with patients, including those with diabetes, effectively according to their needs. Importantly, the educational level of patients might mask their deficiencies in numerical skills, with well-educated people not necessarily having adequate or high health numeracy skills [4].

Several studies have indicated that numeracy has a substantial influence on health conditions and behaviors [5–10], although these effects have not yet been extensively investigated. Low numeracy was associated with increased hospitalization among patients with asthma [5], poor anticoagulation control among patients receiving warfarin therapy [6], and a high body mass index in adult primary care patients [7]. The influence of numeracy on the health of patients with diabetes has been studied in a small number of studies. An association was detected between low diabetes numeracy and poor glycemic control [8], poor diabetes-related knowledge [9], and poor diabetes self-efficacy [10].

Because diabetes education is an important part of treatment [11], there is a need to assess the skills of patients so that educational instructions can be tailored accordingly. Poor numeracy in patients with diabetes is not easy to detect and can be left unrecognized [12]. Therefore, it is important to have an assessment tool to measure such a critical skill for diabetes care. A number of validated tools exist to measure numeracy alone or as part of a literacy test [13–16]. However, the Diabetes Numeracy Test (DNT) is the only validated test for measuring the numeracy skills required by patients with diabetes [17–19].

To our knowledge, numeracy skills related to diabetes management have never been investigated addressed in Saudi Arabia or in the Arab world. This study focused on validating an Arabic-language version of the most commonly used version of DNT, the 15-item DNT (DNT-15), in order to pave the way for researchers, diabetes educators, and clinicians to improve diabetes management in the Arab world. The 15-item Arabic Diabetes Numeracy Test will be useful for researchers to further explore the effect of diabetes numeracy on the health of patients and for quality assessment of educational services. This simple tool will help healthcare providers to identify individuals in need of additional education or training regarding their self-management.

## Methods

### Participants

Overall 176 adult patients (18 years or older) with Type 1 diabetes were recruited from the Diabetes Clinic at King Fahad National Guard Hospital in Riyadh (S1 File). The clinic chosen provides care for patients with diabetes from different socioeconomic backgrounds. The clinic serves the military personnel and their families as well the employees of a large University system and their dependents. Patients with diabetes entering the clinic were approached and asked to participate in the study (85% response rate). Illiterate patients and those with a disability, such as vision problems, dementia, or psychosis, which would prevent them from participating in the study, were excluded. Written consent was obtained from patients before participation in the study. Data collection continued over a 5 month period.

### Instrument

The original DNT, consisting of 43 items, was designed for English speaking population and initially contained question assessing numeracy skills related to nutrition, exercise, glucose monitoring, medication intake, and insulin use [20]. This assessment tool tests the numerical calculations and interpretations that are needed by patients with diabetes for self-management. A shortened version of the instrument consisting of 15 items (DNT-15) was later recommended for use among English speaking patients with diabetes [17]. This shortened version showed good internal reliability (KR-20 = 0.90).

The first step the validation process was to translate the English-language DNT-15 scale, into Arabic. DNT-15 was translated by two professional translators into classical Arabic. After translation the instrument was reviewed by two bilingual investigators and a diabetic educator to check for appropriateness of the language used. The reviewed version of the DNT-15 was then back translated into English by another translator and compared to the original by two investigators to check for consistency. The final Arabic version of the test was reviewed by a panel of six healthcare providers (two health educators, two dieticians, one primary care physician, one diabetologist), one research investigator, and one math instructor for appropriateness of language (cultural equivalence of the wording), face validity (apparently measuring mathematical ability in patients with diabetes), and content validity. Content validity was evaluated by content validity ratio (CVR) and content validity index (CVI). All discrepancies were corrected as suggested. Translators and healthcare professionals were not familiar with the instrument prior to the validation process. The obtained Arabic DNT-15 was piloted on a sample of patients with diabetes (n = 15) to ensure that all the items used were clear and understandable and to modify any existing ambiguity. Patients were asked to rate the language of the test based on the following five criteria: clarity, simplicity, ambiguity, and time-feasibility. The scale was modified according to the feedback received. The modifications included adding figures to facilitate understanding. The participants in the pilot study were not included in the main study.

Finally a small number of bilingual male and female individuals from the general public (n = 17) were asked to complete both the English and Arabic versions of the scale consecutively on the same day and within ten minutes from the first administration. The Spearman correlation coefficient (r) between the English and the Arabic versions of the test was 0.94 (p<0.001).

Moreover, the validity of the Arabic DNT-15 was assessed by concurrently administering the Arabic version of the Diabetes Knowledge Test (DKT) to all participants in the study. The DKT is a valid and reliable test that was developed by the Michigan Diabetes Research Training Center and initially translated to Arabic and validated by Al-Adsani et al. [19]. The internal reliability of the Arabic DKT using Cronbach α was 0.70. The DKT consists of 23 items; 14 items are general knowledge about diabetes (diet, blood glucose monitoring, self-care, and symptoms and complications) and 9 items are related to insulin use.

### Administration of the instrument

Informed consent was obtained from all participants before the administration of the structured questionnaire that included the Arabic version of the DNT-15, and the Arabic version of the DKT. Items responses were scored as correct or incorrect and final scores were converted to a percentage (range: 0–100). Calculators are usually used with the instrument, and were provided for the participants in the study. Demographic characteristics, such as age, gender, level of education and monthly income were collected. Moreover, participants were asked about the duration of diabetes, medication (insulin only or a combination of insulin and oral medication), comorbidities, and glycemic control. The questionnaires were collected from patients after 30 minutes. Medical records were consulted when necessary to extract information such as confirmation of duration of diabetes and other clinical information. Correlating DNT scores with patients ‘characteristics provided an additional means for assessing validity.

In order to check for reliability of the instrument, a test-retest procedure was applied among 29 participants. Initially, 42 patients were asked to consent for a retest after one week and of those 29 accepted (69% response rate). The instrument was self-administered; however, if a patient was unable to read the questions, the investigator in the clinic read the questions to him/her and documented the responses. The study was approved by the Institutional Review Board at King Saud Bin Abdulaziz University for Health Sciences and King Abdullah International Medical Research Center.

### Data analysis

Descriptive statistics (means with standard deviations and frequency distributions) were calculated for age, education, and reported income. The chi-square test was used to determine any possible association between parameters when applicable. The percentages (%) of responses were calculated for all items of the scale. The scale was scored according to the number of correct items for each participant, which was converted to a percentage. Correct responses for the DNT-15 earned a full point; wrong or missing answers were scored at zero. There were 15 questions, for a total of 15 points with increasing values indicating higher numeracy skills (% range: 0–100). The same scoring system was also applied for the DKT. To test validity of the instrument, bivariate relationship were assessed between the Arabic DNT-15 scores and questionnaire variables, and the Arabic version of the DKT scores using Spearman correlation coefficient (r). The mean CVR and mean CVI were calculated for the Arabic DNT-15 as indicators of content validity. The internal consistency reliability and the test-retest reliability were assessed using Kuder-Richardson Formula 20 (KR-20) and Spearman’s Correlation Coefficient (r). Stata12 software (College Station, Texas) was used for all analyses. All analyses were considered significant at alpha = 0.05.

## Results

The mean age of the 176 participants with diabetes was 24.7 years with a standard deviation of 9.3 years. The majority of patients were female (79.5%), under the age of 40 years (89.8%), and with a monthly income of no more than 11 thousand Saudi riyals (85.6%). Forty-six percent of the respondents were educated to high-school level or above. Only 17 patients (9.7%) were receiving a combination of oral anti-diabetic drugs and insulin. Most of the patients (72.2%) had had the disease for more than 5 years. Uncontrolled HbA1c levels of more than 7% were present in the vast majority of patients (94.3%). Slightly more than one-third of patients (34.1%) had comorbidities; Diabetic retinopathy was the most frequent (17.6%) comorbidity, followed by diabetic neuropathy (6.3%), cardiovascular disease (3.4%), and nephropathy (3.4%). Other health problems were present in 15.9% of the patient population, including cancers, thyroid diseases, urinary tract infections, hypertension, and hypercholesterolemia. Approximately 14% of patients had two or more comorbidities. Characteristics of participants are presented in Table 1. The average score (±standard deviation) for the Arabic DNT-15 was 53.3±18.7. The topics of nutrition and exercise were confined in a domain in the scale that received the lowest average percentage of correct answers (44.5%), whereas this percentage was higher for the medication (54.8%) and blood glucose monitoring (60.8%) domains. The lowest correct responses were for questions 13 and 14 about multistep insulin management (33% and 36.9%; respectively). Both questions required patients to interpret a word problem and use multi-step mathematics to determine the correct insulin dosage. Also, questions 1 and 2 dealing with calculating carbohydrates and food labels were not answered accurately respectively by 63.1% and 60.8% of the participants. **Table 2** is a description of the DNT-15 items and a presentation of the sample performance on each item with the corresponding numeracy skills related to diabetes management.

**Table 1:** Characteristics of study sample (N = 176).

Variable	n	%
		
Age (years) (μ = 24,7;SD = ±9.3)		
**<40**	158	89.8
**≥40**	18	10.2
Gender		
**Male**	36	20.5
**Female**	140	79.5
Education		
**≤ High School**	94	53.4
**>High School**	81	46
Monthly Income (SAR)*		
**≤ 11,000**	137	85.6
**>11,000**	23	14.4
Duration of DM** (years)		
**≤5**	49	27.8
**>5**	127	72.2
Treatment of DM		
**Insulin only**	159	90.3
**OADs and Insulin**	17	9.7
HbA1c***		
**Controlled ≤7%**	10	5.7
**Uncontrolled >7%**	164	94.3
Comorbidities		
**Cardiovascular**	6	3.4
**Nephropathy**	6	3.4
**Neuropathy**	11	6.3
**Eye problems**	31	17.6
**Others**	28	15.9
No. of comorbidities		
**One**	36	20.5
**Two**	15	8.5
**Three or more**	9	5.1

*SAR = Saudi Riyals (1SAR≈0.27 US Dollars)

**DM = Diabetes Mellitus

***Glycated HaemoglobinA1C

**Table 2:** Description of domains, items, and distribution of correct answers for the Diabetes Numeracy Test (DNT-15) (N = 176).

Domain (item)	Math Problem Type	Answered Correctly n(%)
*Nutrition and Exercise (item 1–4)*		
**1.Calculate Carbohydrates using food label**	Multiplication/Division	65(36.9)
**2.Calculate Carbohydrates using food label**	Fraction/Decimal	69(39.2)
**3.Calculate Carbohydrates using food label**	Fraction/Decimal	98(55.7)
**4.Exercise Management**	Multi-step Mathematics	81(46)
*Blood Glucose Monitoring (item 5–6)*		
**5.Recognize normal glucose range**	Numeration/Counting/Hierarchy	96(54.5)
**6.Calculate number of test strips needed for travel**	Multiplication/Division	117(66.5)
**7.Test strip management**	Time Management	108(61.4)
*Medication (item 8–15)*		
**8.Oral Medication and time management**	Addition/Subtraction	137(77.8)
**9.Insulin dosing using insulin: carbohydrates ratio**	Multiplication/Division	118(67)
**10.Sliding scale insulin use**	Numeration/Counting/Hierarchy	116(65.8)
**11.Carbohydrates counting, multistep insulin management**	Multi-step Mathematics	81(46)
**12.Carbohydrate counting, multistep insulin management**	Multi-step Mathematics	114(64.8)
**13.Multistep insulin management**	Multi-step Mathematics	65(36.9)
**14.Multistep insulin Management**	Multi-step Mathematics	58(33)
**15. Insulin dosing using syringe with analog scale**	Numeration/Counting/Hierarchy	83(47.2)

Kruder-Richardson (KR-20) for assessing internal consistency reliability for the Arabic DNT-15 was good (0.8). The test-retest reliability of the Arabic DNT-15 was moderately high with Spearman Correlation coefficient of 0.75.

As for the evaluation of validity, the Arabic DNT-15 scores exhibited significant positive correlation with, age (r = 0.175, P<0.05), education (r = 0.198, p<0.05), and monthly income (r = 0.271, p<0.001) and the Knowledge scores (r = 0.42, p <0.01). By contrast, a negative significant correlation was found between HbA1c level and the DNT-15 total scores (r = -0.158, p<0.05). As such, individuals of an older age or with higher education, higher income, more diabetes knowledge, or a lower HbA1c level were more likely to have higher DNT-15 total scores (**Table 3**). Content validity results from the expert panel showed a mean Content Validity Ratio (CVR) of 0.75 and a mean Content Validity Index (CVI) of 0.89.

**Table 3:** Arabic Diabetes Numeracy Test (DNT-15) Spearman correlation with different variables.

	Arabic DNT-15 Total Score	
	r	p-value
**Age**	0.17	0.02
**Education**	0.2	0.009
**Income**	0.27	0.001
HbA1c*	-0.16	0.04
DKT score**	0.42	<0.001
**Duration of Diabetes**	0.12	0.2

*Glycated Hemoglobin A1C.

**Diabetes Knowledge Test score.

## Discussion

This is the first study designed to evaluate the reliability and validity of the Arabic DNT. The Arabic translation of the DNT-15 was found to have good numerical reasoning testing properties among insulin using patients with diabetes. The reliability of the scale was good, with a KR-20 of 0.8, which is slightly lower than that of the English version of the test (DNT-15; KR-20 = 0.90) [17] and close to that of the Latino version (DNT-15 Latino; KR-20 = 0.78) [21].This reliability is consistent with those of other health numeracy scales (KR-20 = 0.56–0.80) [4,22].

The Arabic DNT-15 score was significantly correlated with age (ρ = 0.17), education (ρ = 0.198), income (ρ = 0.271), glycemic control (ρ = 0.158), and DKT score (ρ = 0.423). Similarly, the score of the English version of the scale has been shown to be significantly correlated with education (ρ = 0.52), income (ρ = 0.51) and DKT score (ρ = 0.71) [17]. The DNT-15 Latino score has been shown to be significantly correlated with education (ρ = 0.36) and income (ρ = 0.27) but not with glycemic control (ρ = 0.064) [21].

We expected the Arabic DNT-15 score to be positively correlated with duration of diabetes. The results showed higher scores in respondents with more years of disease (>5 years since diagnosis) than in other patients, but the relationship between score and disease duration was not significant. The same finding was also observed in a study of Cavanaugh K et al. where numeracy was not significantly associated with duration of diabetes [9]. Moreover, no relationship was found between the Arabic DNT-15 score and the presence of comorbidities. Despite the observation that sex was not significantly associated with the Arabic DNT-15 score, the mean score among female patients was slightly higher than that among male patients. Sex was not significantly associated with DNT-15 scores in a previous study from North America by Zaugg et al. [23] The mean Arabic DNT-15 score in the sample we studied was only 53% of correct answers, although nearly half the patients had received higher formal education (high school or higher), and all had attended at least one diabetes education session prior to testing. This mean score was lower than the mean DNT score previously reported in adult patients with type 1 and type 2 diabetes, which was 61% of correct answers [17]. The performance of respondents in the Latino version of the score (DNT-15 Latino) was far worse than that in our study, with only 26% of correct answers on average [21]. Although the performance of the highly educated participants in other studies was better than that of the less educated participants, they nevertheless had poor diabetes numeracy skills [4,17].

Calculating carbohydrates intake using food labels and the multi-step management of insulin therapy were the most problematic areas in the scale, with the lowest frequencies of correct answers. These items of the scale require higher skills of multi-step mathematics than other items. Similar findings were reported in the study in which the DNT-15 was assessed [17] and the Latino version of the test was assessed [21]^.^

The topics of nutrition and exercise were confined in a domain in the scale that received the lowest average percentage of correct answers (44.5%), whereas this percentage was higher for the medication (54.8%) and blood glucose monitoring (60.8%) domains.

There are several possible explanations for this result. The domain of nutrition and exercise requires more advanced math skills than the blood glucose monitoring domain, which suggests that the type of math problems for each domain might explain the observed differences. However, the math skills required to perform well in the nutrition and exercise domain are less advanced than those required for the domain of medication, for which the higher average percentage of correct answers was higher than that for the nutrition and exercise domain. A different explanation for the observed results is that patients with diabetes generally show greater interest in information about medications and blood glucose monitoring than in that about nutritional requirements or exercise. This difference in interest between medication and self-care could be one explanation for the prevalence of diet-related chronic diseases as well as the lack of physical activity in Arab countries, and which is compounded by the fact that the health authorities have historically focused more attention on treatment than prevention [24].

## Conclusions

Patients with diabetes need numeracy skills to manage their disease. Their level of education does not reliably reflect their level of numeracy, and low numeracy in patients might not be noticed by healthcare providers. The availability of a screening tool for use with Arabic-speaking population to identify patients with difficulties in certain diabetes-related numeracy skills is important. Our findings show that Arabic DNT-15 is a valid and reliable scale for this purpose and this is the first such screening tool that is available for this patient population.

We recommend that clinicians and other healthcare providers communicate numerical information in a simple way. We also recommend that the influence of diabetes numeracy on important factors (i.e. informed medical decisions related to risk and benefits of treatments, survival rates, probability of death and disability, medication compliance, and risk communication in general) other than diabetes self-management among Arabic-speaking patients is assessed in future studies.

## Supporting information

S1 File This is the S1 data file. (PDF)
