# Supplementary material for: Validation of a diabetes numeracy test in Arabic
Source: PLoS One. 2017 May 4;12(5):e0175442. doi: 10.1371/journal.pone.0175442 (PMC5417435; doi:10.1371/journal.pone.0175442)
Supplement: S1 File — (PDF) [file pone.0175442.s001.pdf]

dkt1	dkt2	dkt3	dkt4	dkt5	dkt6	dkt7	dkt8	dkt9
1	0	0	0	0	0	1	0	0
0	0	0	0	0	0	0	1	0
1	1	1	0	1	1	1	1	1
1	0	0	0	0	1	0	0	0
1	0	0	0	0	0	0	0	1
1	1	0	0	1	0	1	1	1
1	1	1	0	0	0	0	1	1
0	0	0	0	1	0	0	0	1
0	1	0	0	0	0	0	1	1
0	1	0	1	1	1	1	1	1
1	0	0	0	0	1	0	0	1
0	0	0	0	0	0	0	1	1
0	0	1	0	0	1	1	0	1
1	0	0	1	0	0	0	0	0
0	0	0	0	1	1	0	1	1
1	0	0	0	0	0	1	0	1
1	1	1	1	1	1	1	0	1
1	0	0	1	0	1	0	0	1
1	0	0	1	1	1	1	1	1
1	0	1	0	0	1	1	1	1
1	1	0	0	1	1	0	1	1
1	1	0	0	1	0	0	0	1
1	0	0	0	1	1	1	0	1
0	1	0	0	0	1	1	1	1
1	0	0	0	1	1	1	0	1
1	1	0	0	1	1	1	1	1
1	0	0	0	1	1	1	0	1
1	1	0	0	1	1	0	0	0
1	0	0	0	0	0	1	0	1
1	0	0	0	0	1	0	1	1
1	0	0	0	1	0	0	0	1
1	1	0	0	1	0	1	1	0
0	1	0	0	0	0	0	0	0
0	0	0	0	1	1	1	0	1
1	1	0	0	0	1	0	0	1
1	0	0	1	1	1	0	1	1
0	0	0	1	1	1	1	0	1
1	0	0	0	1	1	0	0	1
1	0	0	0	0	0	0	0	1
1	0	0	0	0	1	1	1	1
1	0	0	0	0	1	0	1	1
0	1	0	0	1	1	1	1	1
1	0	1	0	1	1	1	1	1
1	0	0	0	1	1	1	1	1
1	1	1	0	1	0	0	1	1
1	0	1	1	0	1	0	1	1

1	0	1	0	1	1	1	0	1
1	0	0	0	1	0	1	0	1
1	1	0	0	1	1	0	1	0
1	0	0	0	1	0	0	0	1
1	0	1	0	0	1	0	1	1
1	1	0	1	1	0	1	0	1
1	0	0	1	0	1	0	0	1
0	1	0	0	0	1	0	1	1
1	0	0	0	1	0	1	1	1
1	0	0	0	1	0	1	0	1
1	0	0	0	0	0	0	0	1
1	0	1	0	0	1	1	0	1
1	1	1	0	1	1	1	1	1
1	1	0	1	0	1	1	0	1
0	0	0	0	1	1	1	1	1
1	0	0	0	0	1	1	0	1
0	0	0	0	0	0	0	0	1
1	1	0	1	0	1	1	1	1
1	0	0	1	1	0	1	0	1
1	0	0	0	0	1	0	1	1
0	0	0	1	1	0	0	0	1
1	1	1	0	0	1	0	0	0
1	1	0	0	0	0	1	1	1
0	0	0	0	1	1	0	1	1
1	1	0	0	0	1	0	0	1
1	1	0	0	0	1	1	1	1
0	0	0	0	0	1	0	0	0
1	0	0	0	0	1	1	0	1
0	0	0	0	1	1	0	0	1
1	1	1	1	0	1	0	1	1
1	1	1	0	0	0	0	1	1
0	0	0	1	1	1	0	1	1
1	0	0	0	0	0	0	1	1
1	1	0	0	0	0	1	0	1
1	0	0	0	1	1	0	0	1
1	1	0	0	1	1	0	0	1
1	1	0	1	1	1	0	1	1
0	0	0	0	0	0	1	0	1
1	1	0	0	0	0	0	1	1
1	1	1	1	1	1	0	1	1
1	0	0	0	1	1	0	0	1
1	0	0	0	0	1	0	0	1
0	1	0	0	0	0	0	1	1
0	1	1	0	1	0	0	1	1
1	1	0	1	1	1	0	0	1
1	1	0	0	1	1	0	0	1
0	0	1	0	1	1	1	0	1

0	1	0	0	1	1	1	0	1
1	1	0	0	0	0	0	0	1
1	1	0	1	1	1	1	1	1
0	1	0	0	1	1	0	0	1
1	0	0	0	0	1	0	0	1
0	0	0	0	1	1	0	1	1
0	1	0	1	1	0	0	0	1
1	0	0	0	1	0	0	0	0
1	0	0	0	0	1	0	1	1
1	1	0	1	1	1	0	1	1
1	0	0	0	1	1	0	0	1
1	1	0	0	1	0	1	1	0
1	0	0	0	1	1	0	1	1
1	1	0	0	1	1	0	0	1
0	1	0	0	1	0	0	0	1
1	0	0	0	0	1	0	1	1
1	1	0	0	1	0	1	1	1
1	0	0	0	0	1	0	0	1
1	0	1	0	0	1	1	1	1
1	0	0	0	0	0	0	1	1
1	0	0	0	1	0	1	1	1
1	1	0	1	0	1	1	1	1
1	1	0	0	1	0	0	1	1
1	1	1	1	0	1	1	1	1
1	0	0	0	1	1	0	0	1
1	1	0	0	1	1	1	0	1
1	0	1	0	1	1	1	0	1
1	0	0	0	1	0	1	1	1
1	1	1	0	0	0	0	1	1
1	0	0	0	0	1	0	0	0
0	0	0	0	1	1	0	0	1
0	1	0	0	0	0	0	1	1
1	0	0	0	0	0	0	0	0
0	0	0	0	1	1	1	0	1
1	0	0	0	0	0	1	0	1
1	0	1	1	0	1	0	1	1
1	0	0	0	1	1	0	1	1
0	1	0	0	0	1	0	0	1
0	0	0	1	0	1	0	0	1
1	0	0	1	1	1	1	1	1
1	0	0	0	1	1	0	0	1
1	1	1	1	0	1	1	0	1
1	0	0	1	1	1	0	1	1
0	0	0	0	1	1	0	1	1
1	1	0	1	1	1	0	0	0
0	1	0	0	1	0	0	0	1
1	0	0	0	1	0	0	1	1

1	1	1	1	0	1	0	0	1
1	1	0	0	0	0	1	1	1
1	1	1	1	0	0	1	1	1
1	1	0	0	0	0	0	0	0
1	1	1	1	1	1	1	1	1
0	0	0	0	1	1	1	1	1
1	0	0	0	1	0	1	1	1
1	1	0	0	0	1	0	0	0
0	0	1	0	1	1	1	1	1
1	0	0	1	1	1	1	1	1
0	0	1	0	0	1	1	0	1
1	1	0	0	1	1	0	0	0
1	0	0	1	1	1	0	0	1
1	1	0	0	1	1	1	0	1
1	0	0	0	1	1	0	0	1
1	0	1	0	0	1	0	1	1
0	0	0	0	0	1	1	0	0
0	1	1	1	0	1	1	0	0
0	0	1	0	0	0	0	1	1
1	1	0	0	1	0	1	0	1
1	0	0	0	0	1	1	1	0
1	0	1	0	0	0	0	1	1
1	0	1	0	1	1	0	0	1
0	1	0	1	1	0	0	0	0
1	0	1	0	1	1	0	1	1
1	0	0	1	0	0	0	0	0
0	0	0	0	0	1	1	0	1
0	0	0	0	0	0	0	0	1
1	0	0	0	0	0	0	0	1
1	0	0	1	1	1	0	0	1
0	1	0	0	1	1	1	0	1
0	1	0	0	0	1	1	0	1
1	0	0	0	0	1	0	0	0
1	0	0	1	0	1	1	0	1
1	0	0	0	0	0	0	1	0
0	0	0	0	1	0	1	1	1

dkt10	dkt11	dkt12	dkt13	dkt14	dkt15	dkt16	dkt17	dkt18
0	1	1	1	1	0	1	0	1
1	1	1	1	1	1	1	0	0
1	1	1	1	1	1	1	0	1
1	1	1	1	1	1	0	0	1
0	1	0	1	0	0	1	1	1
0	1	1	0	1	0	0	0	1
1	1	1	1	1	1	1	0	1
0	1	1	1	0	1	1	0	1
1	0	1	1	0	0	1	0	1
1	1	1	1	1	1	1	0	0
1	0	0	0	1	1	1	1	1
1	1	1	1	1	0	1	0	0
0	0	1	0	1	1	1	0	1
1	0	1	0	1	0	1	0	0
1	1	1	1	1	1	1	1	1
1	1	1	0	0	0	1	0	1
1	1	1	1	1	1	1	1	1
1	1	1	1	0	1	1	0	1
1	1	1	1	1	1	1	1	1
1	1	1	1	1	0	1	1	0
1	1	1	1	1	1	1	1	1
0	0	1	0	0	0	1	0	0
1	1	1	1	1	1	1	1	1
1	1	1	1	1	0	1	1	1
1	0	1	1	0	1	1	1	0
1	0	1	1	1	1	1	1	1
0	1	1	1	1	0	0	0	0
1	1	1	1	1	1	1	1	1
0	1	0	0	1	0	0	0	1
1	1	0	1	0	1	1	0	0
1	1	1	0	1	0	1	0	1
1	1	1	1	1	1	1	1	1
1	0	0	0	1	0	1	0	1
1	0	1	0	1	1	1	0	1
0	1	1	1	1	0	0	0	1
1	1	1	1	1	0	1	0	0
1	1	1	1	1	1	1	0	1
1	1	1	1	1	1	1	1	0
1	1	1	1	1	1	1	0	0
1	1	1	0	1	0	1	1	0
1	0	1	0	0	0	1	1	1
1	1	1	1	1	1	1	1	1
1	1	1	1	1	1	1	1	1
1	1	1	1	1	0	1	0	1
1	1	1	1	1	1	1	0	1
1	1	1	1	1	1	1	1	1

1	1	1	1	1	1	1	1	1
1	1	1	1	1	1	1	1	1
1	1	1	1	1	1	0	0	0
1	1	1	1	1	0	1	1	0
0	1	1	1	1	0	1	0	0
1	1	1	1	1	1	1	0	1
1	1	1	0	1	0	1	0	1
1	1	1	0	1	1	1	1	0
1	1	1	1	1	1	1	1	1
1	1	1	1	1	1	1	0	1
0	1	0	1	1	0	0	1	0
0	0	1	1	1	0	0	0	0
1	1	1	1	1	0	1	1	0
1	1	1	1	1	1	1	1	1
1	1	1	1	1	1	1	1	1
1	1	1	1	1	1	1	1	1
1	0	0	0	0	1	0	1	0
1	1	1	1	1	1	1	0	1
1	1	1	0	1	1	1	0	1
1	1	1	1	0	1	1	1	0
1	1	1	1	1	0	1	1	1
0	1	0	1	0	0	0	1	1
1	1	1	1	1	1	1	1	1
1	1	1	1	1	0	0	1	1
0	1	0	1	1	0	0	0	1
1	1	1	1	1	1	1	0	0
1	1	1	1	1	0	1	0	0
1	0	1	1	1	1	1	0	0
1	1	1	1	1	1	1	0	1
1	1	1	1	1	0	1	0	1
1	1	1	1	1	0	1	1	0
1	1	1	1	1	1	1	0	1
1	1	0	0	0	0	1	1	1
1	1	1	1	1	1	1	1	0
1	1	1	1	0	0	0	1	1
0	1	1	0	1	0	1	0	0
1	1	1	1	1	1	1	1	1
1	1	1	0	1	1	1	1	1
1	1	1	1	1	0	1	1	0
1	1	1	1	1	1	1	0	0
1	1	1	0	0	0	1	1	1
1	1	1	1	1	1	1	0	1
1	1	1	1	1	1	1	0	0
1	1	1	1	1	0	0	1	0
1	0	1	0	1	0	1	0	0
1	1	1	1	1	1	0	1	1
0	0	1	1	1	1	0	0	0

1	1	1	1	1	1	1	1	0
1	1	1	1	1	1	1	1	0
0	1	1	1	1	0	1	1	0
1	1	1	1	1	1	1	1	0
1	1	1	1	1	1	1	1	0
1	1	0	1	1	1	1	1	0
0	1	1	1	1	0	1	1	1
1	1	0	0	1	1	1	1	1
1	0	1	1	1	1	1	0	0
1	1	1	1	1	1	1	1	0
1	0	1	1	0	0	0	0	0
1	1	0	1	1	0	1	0	0
1	1	1	0	1	1	1	1	0
1	1	1	1	1	0	0	0	1
1	1	1	1	1	1	1	1	0
1	1	1	1	1	1	1	0	1
1	0	1	1	1	0	1	0	0
1	0	1	1	0	0	0	0	0
0	1	1	0	0	0	1	0	1
1	0	1	1	1	1	1	1	1
1	1	1	1	1	1	1	1	1
1	1	1	1	1	0	1	1	1
1	1	0	0	0	0	1	0	0
1	1	1	1	1	1	1	1	1
1	1	0	0	0	1	1	1	1
1	1	1	1	1	1	1	0	1
1	1	1	1	1	1	1	1	1
1	1	1	1	1	1	1	0	1
1	1	1	1	1	0	1	0	1
1	1	0	1	1	0	1	1	1
1	1	1	1	1	1	1	1	0
1	1	0	0	1	1	1	1	1
1	1	0	1	1	1	1	1	1
1	1	1	1	1	1	1	1	1
1	1	1	1	1	1	1	1	1
1	1	1	1	1	1	1	1	0
1	1	1	0	1	1	1	0	1
1	1	1	1	1	1	0	0	0
1	1	1	1	1	0	1	0	1
1	1	1	1	1	1	1	1	1
0	0	1	1	0		0	1	1
1	1	1	1	1	1	1	1	1
1	1	1	0	1	1	1	0	1
1	1	1	1	1	0	0	0	0
0	1	1	1	1	0	1	0	1
1	1	1	0	1	1	1	0	1
0	1	0	1	0	1	0	1	1

1	1	1	1	1	1	0	1	1
1	1	0	1	1	0	1	1	1
1	1	1	1	1	0	1	0	1
1	1	0	1	1	0	1	0	1
1	1	1	1	1	1	1	1	1
1	1	1	0	1	1	0	0	1
0	0	1	0	1	1	1	0	1
1	1	1	1	1	0	1	1	0
1	1	1	1	1	1	1	1	1
1	1	1	1	1	1	1	0	1
0	0	1	1	1	1	1	1	1
1	1	0	0	0	0	1	0	0
1	1	1	1	1	1	1	0	1
1	1	1	1	1	1	1	0	1
0	1	1	1	1	0	1	0	1
1	1	1	1	1	1	1	1	1
1	0	1	1	1	0	1	0	1
0	1	1	0	1	0	0	1	1
1	0	0	0	0	0	0	0	1
0	1	1	1	1	1	1	1	0
1	1	1	1	1	1	0	0	1
1	1	1	1	1	0	1	1	1
1	1	1	1	1	1	1	1	1
1	1	1	1	1	0	1	1	1
1	1	1	1	1	1	1	1	1
1	1	1	0	1	1	1	1	1
1	1	0	0	1	0	0	0	1
1	0	1	1	0	0	1	0	1
1	1	1	1	1	1	1	0	1
1	1	1	1	0	0	0	0	1
1	1	1	1	1	0	1	1	1
1	1	0	0	0	1	1	0	1
0	1	1	0	0	0	0	1	0
0	1	1	0	1	0	1	0	1
1	1	0	1	1	1	1	0	0
1	1	1	1	1	0	1	0	0

dkt19	dkt20	dkt21	dkt22	dkt23	age	gender	education	budget
1	1	1	0	0	23	1	2	1
1	1	1	1	1	17	2	2	4
1	1	1	1	1	22	1	3	3
1	1	1	1	1	19	1	3	2
1	1	1	1	1	20	1	2	2
1	1	1	1	1	28	1	3	3
1	1	1	1	0	18	2	2	1
1	1	1	1	1	23	2	2	1
1	1	1	0	0	25	2	3	1
1	1	1	1	1	28	2	3	2
1	1	1	1	0	16	2	2	3
1	1	1	1	1	16	1	2	1
1	1	1	0	1	30	2	1	1
1	0	1	0	1	20	2	3	2
1	1	1	1	1	20	2	3	5
1	1	1	1	1	29	2	2	
1	1	1	1	1	20	2	2	2
1	1	0	1	1	20	2	2	2
1	1	1	1	1	30	2	2	2
1	1	1	1	1	21	2	3	2
1	1	1	1	1	17	2	1	3
1	1	1	1	0	21	2	3	2
1	1	1	1	1	16	2	2	5
1	1	1	0	0	38	2	3	1
0	0	1	1	0	35	2	1	1
1	1	1	1	1	19	1	3	1
0	0	0	0	0	23	2	3	1
1	1	1	1	1	19	2	2	2
0	0	0	1	0	19	2	3	1
1	1	1	1	1	19	2	2	2
1	1	1	1	1	27	2	2	1
1	1	1	1	1	18	2	2	1
0	0	1	1	0	18	1	2	
1	1	1	1	1	20	2	3	2
1	1	1	0	1	22	2	3	2
1	1	1	1	1	46	2	1	2
1	1	1	1	1	15	2	1	5
1	1	1	1	1	25	2	2	1
1	1	1	1	1	24	2	1	1
1	1	1	1	0	37	2	1	2
1	0	1	0	0	20	2	2	1
1	1	1	1	1	18	2	2	2
1	1	1	1	0	29	2	3	2
1	1	1	1	0	19	2	3	5
1	1	1	1	1	30	2	2	1
1	1	1	1	1	25	2	2	3

1	1	1	1	1	21	2	3	1
1	1	1	1	1	21	2	3	2
1	1	1	0	1	23	2	3	2
1	1	1	1	1	16	2	2	1
1	1	1	1	1	25	1	2	1
1	1	1	1	1	50	2	3	1
1	1	1	1	1	60	1	1	4
1	1	1	1	0	20	2	2	1
1	1	1	1	1	16	2	2	4
1	1	1	1	1	27	2	3	2
1	1	1	1	1	17	2	2	3
1	1	1	0	1	18	2	3	1
1	0	1	1	0	43	2	1	2
1	1	1	1	1	21	2	3	3
1	1	1	1	1	40	2	3	3
1	1	1	1	1	35	2	3	3
1	1	1	1	0	23	2	2	3
1	1	1	0	1	20	2	3	
1	1	1	1	1	17	1	2	4
1	1	1	1	1	18	2	2	1
1	1	1	1	1	20	2	3	3
1	0	0	0	0	27	2	3	1
1	1	1	1	0	34	2	3	3
1	1	1	1	0	19	2	3	1
1	1	1	0	0	19	2	3	2
1	1	1	1	1	28	2	2	2
1	1	1	1	1	22	2	2	1
0	1	1	1	1	20	2	2	
1	1	1	1	1	19	1	3	1
1	1	1	1	0	25	2	3	1
1	0	1	1	1	25	2	2	1
1	1	1	1	1	27	2	3	2
1	0	1	1	0	16	2	2	3
1	1	1	1	1	22	2	3	1
1	1	1	1	1	52	2	2	3
1	1	1	1	0	20	1	2	1
1	1	1	1	0	44	2	3	4
1	1	1	1	1	23	2	3	1
0	1	1	1	1	26	2	3	1
1	1	1	1	1	26	2	2	3
1	1	1	1	0	25	2	3	2
1	1	1	0	1	22	2	3	2
1	0	1	1	1	50	2	1	
1	1	1	1	1	26	2	3	
1	1	1	1	1	18	2	3	3
1	1	1	1	1	33	2	3	2
1	1	1	1	1	44	2	1	1

1	1	1	1	1	22	2	2	1
1	1	1	1	1	17	2	2	2
1	1	1	1	1	45	2	3	4
1	1	1	1	1	25	2	2	3
1	1	1	1	1	28	2	3	2
1	1	1	1	1	17	2	2	2
1	1	1	1	1	22	2	3	3
1	1	1	0	1	25	2	3	3
1	1	1	1	1	16	1	2	4
1	1	1	1	1	50	2	3	2
1	1	1	1	1	16	1	1	1
1	1	1	1	0	17	1	2	1
1	1	1	1	1	42	2	3	1
1	1	1	1	1	24	2	2	1
1	1	1	1	1	24	2	2	1
1	1	1	1	1	26	2	3	2
1	1	1	1	1	20	2	3	
1	1	1	0	0	19	1	2	1
1	1	1	0	1	17	2	2	
1	1	1	1	0	16	1	2	2
1	1	1	1	1	16	2	2	5
1	1	1	1	1	18	2	3	5
1	1	1	1	1	16	2	1	3
1	1	1	1	1	20	2	3	4
1	1	1	1	1	18	2	2	2
1	1	1	1	1	20	1	3	3
1	1	1	1	1	22	2	3	1
1	1	1	1	1	26	2	3	5
1	1	1	1	1	25	2	3	
1	1	1	1	0	21	2	3	1
1	1	1	1	1	23	2	2	2
1	1	1	1	0	18	2	2	1
1	1	1	1	1	18	2		4
1	1	1	1	1	22	2	3	1
1	1	1	1	1	40	2	3	3
1	1	1	1	1	49	2	1	1
1	1	1	1	0	35	2	1	1
0	0	0	1	0	29	2	2	2
1	1	1	1	1	16	2	2	
1	1	1	1	1	22	2	3	4
0	1	0	0	1	18	2	2	
1	1	1	1	1	54	2	3	5
1	1	1	1	1	50	2	2	2
1	1	1	1	0	19	2	3	1
1	1	1	1	0	43	1	3	5
1	1	1	1	1	15	2	2	3
1	1	1	1	1	16	1	1	3

1	1	1	1	1	34	2	2	1
1	1	1	1	1	28	2	3	3
1	1	1	1	1	28	2	1	2
1	1	1	1	1	17	1	2	2
1	1	1	1	1	25	2	3	2
1	1	1	1	1	16	1	2	3
1	1	1	1	1	19	2	2	2
1	1	1	1	1	57	2	3	5
1	1	1	1	1	16	1	2	5
1	1	1	1	1	21	2	3	5
1	1	1	1	1	18	1	2	1
1	1	0	1	1	14	1	2	
1	1	1	1	1	21	2	3	1
1	1	1	1	1	22	2	3	1
1	0	1	0	0	21	2	3	2
0	0	1	1	1	16	2	2	2
1	1	1	1	1	18	2	3	
1	1	1	1	1	25	1	2	2
0	1	1	1	1	19	1	2	
1	1	1	1	1	34	1	3	3
1	1	1	1	1	31	1	3	3
1	1	1	1	1	20	2	3	4
1	1	1	1	1	16	2	1	
1	1	1	1	1	22	1	2	2
1	1	1	1	1	20	2	2	2
1	0	1	0	0	29	2	2	1
1	0	1	0	1	25	2	1	2
1	0	0	1	1	28	1	2	2
1	1	1	1	1	21	2	2	
1	1	1	1	1	20	1	3	1
1	1	1	1	0	27	2	3	1
1	1	1	0	0	23	1	3	2
0	1	0	0	0	25	1	2	2
1	1	1	1	1	22	2	3	2
0	0	0	0	0	20	2	2	2
0	0	1	1	1	24	1	3	1

dmduratior	txtype	hba1c	none	cvd	nephropatl	eyeprobler	neuropathy	others
2	4	3	1	0	0	0	0	0
2	4	3	1	0	0	0	0	0
1	4	2	1	0	0	0	0	0
2	4	3	1	0	0	0	0	0
2	4	3	1	0	0	0	0	0
2	4	2	1	0	0	0	0	0
2	4	3	1	0	0	0	0	0
2	4	3	1	0	0	0	0	0
1	4	2	1	0	0	0	0	0
3	4	2	1	0	0	0	0	0
1	4	3	1	0	0	0	0	0
1	4	3	1	0	0	0	0	0
2	4	2	1	0	0	0	0	0
2	4	3	1	0	0	0	0	0
2	4	2	1	0	0	0	0	0
3	4	3	0	1	0	0	0	1
2	4	2	1	0	0	0	0	0
2	4	3	0	0	0	1	0	0
1	4	2	1	0	0	0	0	0
1	4	2	1	0	0	0	0	0
1	4	3	1	0	0	0	0	0
1	4	3	1	0	0	0	0	0
2	4	3	1	0	0	0	0	0
1	4	1	1	0	0	0	0	0
2	3	3	0	0	0	1	0	1
2	4	2	1	0	0	0	0	0
1	4	2	1	0	0	0	0	0
1	4	3	0	0	1	0	0	1
1	4	3	1	0	0	0	0	0
2	4	3	1	0	0	0	0	0
2	4	2	1	0	0	0	0	0
2	4	1	0	0	0	1	0	0
2	4	3	1	0	0	0	0	0
2	4	3	1	0	0	0	0	0
2	4	3	1	0	0	0	0	0
2	3	3	0	0	0	1	0	1
2	4	3	1	0	0	0	0	0
2	4	3	0	0	0	0	1	1
2	4	3	1	0	0	0	0	0
2	3	3	0	0	0	0	1	1
1	4	3	1	0	0	0	0	0
2	4	3	0	0	0	0	0	1
2	4	3	1	0	0	0	0	0
2	4	2	0	0	0	0	0	1
3	4	3	0	0	0	1	0	0
2	4	3	0	0	0	0	0	1

2	4	3	0	0	0	1	0	0
2	4	3	1	0	0	0	0	0
3	4	2	0	0	0	1	0	1
2	4	3	0	1	1	1	0	0
1	4	3	0	0	0	0	0	1
2	4	2	0	0	0	0	0	1
3	3	3	0	0	0	1	1	1
1	4	2	1	0	0	0	0	0
2	4	3	1	0	0	0	0	0
2	4	2	1	0	0	0	0	0
2	4	3	1	0	0	0	0	0
1	4	2	1	0	0	0	0	0
2	3	3	0	0	0	0	0	1
2	4	3	1	0	0	0	0	0
2	3	3	0	0	0	1	1	1
3	4	3	0	0	0	0	1	1
3	4	3	1	0	0	0	0	0
2	4	3	0	1	0	0	0	0
1	4	3	1	0	0	0	0	0
2	4	3	1	0	0	0	0	0
2	4	3	1	0	0	0	0	0
2	4	3	1	0	0	0	0	0
2	4	2	0	0	1	1	0	0
2	4	3	1	0	0	0	0	0
1	4	3	1	0	0	0	0	0
3	4	2	1	0	0	0	0	0
2	4	2	1	0	0	0	0	0
2	4	3	1	0	0	0	0	0
2	4	2	1	0	0	0	0	0
1	4	2	1	0	0	0	0	0
2	4	1	1	0	0	0	0	0
3	4	2	0	0	0	1	0	0
1	4	3	1	0	0	0	0	0
2	4	3	1	0	0	0	0	0
3	4	3	0	1	0	0	0	0
1	4	1	1	0	0	0	0	0
2	3	3	0	0	0	0	0	1
3	4	3	0	0	0	0	0	1
1	4	2	1	0	0	0	0	0
3	4	3	0	0	0	1	0	1
1	4	3	1	0	0	0	0	0
2	4	3	1	0	0	0	0	0
3	4	3	0	0	0	0	1	0
2	4	3	0	0	0	0	0	1
1	4	3	1	0	0	0	0	0
3	4	3	0	0	0	1	0	0
3	4	3	1	0	0	0	0	0

2	3	3	0	0	0	0	0	1
2	4	3	0	0	0	1	0	0
3	3	2	0	0	0	0	0	1
2	4	2	1	0	0	0	0	0
2	4	3	1	0	0	0	0	0
1	4	3	1	0	0	0	0	0
2	4	3	0	0	0	0	0	1
3	4	3	1	0	0	0	0	0
2	4	3	1	0	0	0	0	0
2	4	3	0	0	0	1	1	1
1	4	3	1	0	0	0	0	0
1	4	2	1	0	0	0	0	0
1	3	3	0	0	0	1	0	0
2	4	1	0	0	0	1	0	0
1	3	3	1	0	0	0	0	0
2	4	3	0	0	0	0	0	1
2	4	3	1	0	0	0	0	0
2	4	3	1	0	0	0	0	0
1	4	3	1	0	0	0	0	0
2	4	3	0	0	0	1	0	1
2	4	2	1	0	0	0	0	0
2	4	3	1	0	0	0	0	0
2	4	3	1	0	0	0	0	0
2	4	2	0	0	0	1	0	0
2	4	3	0	0	0	1	0	0
1	4	2	1	0	0	0	0	0
2	4	2	1	0	0	0	0	0
3	4	2	0	0	0	1	0	0
2	4	1	1	0	0	0	0	0
2	4	3	1	0	0	0	0	0
2	4	3	0	0	0	0	0	1
2	4	3	1	0	0	0	0	0
2	4	3	1	0	0	0	0	0
2	4	3	1	0	0	0	0	0
1	3	2	1	0	0	0	0	0
3	4		0	1	1	0	0	0
2	3	3	0	0	0	0	0	1
1	4	3	1	0	0	0	0	0
2	4	3	1	0	0	0	0	0
3	4	3	1	0	0	0	0	0
1	4	2	1	0	0	0	0	0
2	3	3	0	0	0	0	1	0
2	3	2	1	0	0	0	0	0
1	4	2	1	0	0	0	0	0
2	4	3	0	0	0	1	1	0
1	4	1	1	0	0	0	0	0
2	4	3	1	0	0	0	0	0

2	4	2	0	0	0	1	0	1
2	4	2	1	0	0	0	0	0
2	4	3	0	0	0	1	0	0
2	4	2	1	0	0	0	0	0
3	4	1	1	0	0	0	0	0
1	4	2	1	0	0	0	0	0
2	4	3	0	0	1	0	0	0
1	3	3	1	0	0	0	0	0
1	4	3	1	0	0	0	0	0
3	4	3	1	0	0	0	0	0
2	4	2	1	0	0	0	0	0
1	4	3	1	0	0	0	0	0
2	4	2	0	0	0	1	0	0
2	4	3	1	0	0	0	0	0
2	4	2	0	0	0	1	0	0
1	4	3	0	0	0	0	1	1
2	4	2	1	0	0	0	0	0
1	4	3	1	0	0	0	0	0
1	4	3	1	0	0	0	0	0
2	4	2	1	0	0	0	0	0
1	4	3	1	0	0	0	0	0
2	4	3	1	0	0	0	0	0
2	4	2	0	0	1	0	0	0
2	4	2	1	0	0	0	0	0
2	4	3	1	0	0	0	0	0
3	4	2	0	0	0	0	1	0
1	4	2	0	1	0	0	0	0
3	4	1	1	0	0	0	0	0
1	4	1	0	0	0	1	0	0
3	4	2	0	0	0	1	0	0
1	3	2	1	0	0	0	0	0
2	4	3	1	0	0	0	0	0
1	4	2	0	0	0	1	0	0
1	4	3	1	0	0	0	0	0
3	4	3	0	0	0	1	0	0
2	4		1	0	0	0	0	0
